# Effects of Physical Exercise on Executive Function in Schizophrenia: Systematic Review and Meta-Analysis

**DOI:** 10.3390/sports13040123

**Published:** 2025-04-16

**Authors:** Nuria Pérez-Romero, Christian Campos-Jara, Caterina Pesce, Sergio Araya Sierralta, Enrique Cerda-Vega, Rodrigo Ramirez-Campillo, Rodrigo Campos-Jara, Cristian Martínez-Salazar, Cristián Arellano-Roco, Victoria Hernández-Cifuentes, Falonn Contreras-Osorio

**Affiliations:** 1 Exercise and Rehabilitation Sciences Institute, Postgraduate, Faculty of Rehabilitation Sciences, Universidad Andres Bello, Santiago 7591538, Chile; nuria.perez@unab.cl; 2 Exercise and Rehabilitation Sciences Institute, Faculty of Rehabilitation Sciences, Universidad Andres Bello, Santiago 7591538, Chile; christian.campos@unab.cl (C.C.-J.); enrique.cerda@unab.cl (E.C.-V.); 3 Department of Movement, Human and Health Sciences, University of Rome “Foro Italico”, 00135 Rome, Italy; caterina.pesce@uniroma4.it; 4 Departamento de Educación Física, Universidad de Atacama, Copiapó 1531772, Chile; sergio.araya@uda.cl; 5 Exercise and Rehabilitation Sciences Institute, School of Physical Therapy, Faculty of Rehabilitation Sciences, Universidad Andres Bello, Santiago 7591538, Chile; rodrigo.ramirez@unab.cl (R.R.-C.); v.hernandezcifuentes@uandresbello.edu (V.H.-C.); 6 Servicio de Psiquiatría, Hospital Mauricio Heyermann, Angol 4650207, Chile; drodrigo.campos@gmail.com; 7 Department of Physical Education, Sports and Recreation, Pedagogy in Physical Education, School of Education and Social Sciences and Humanities, Universidad de La Frontera, Temuco 4811230, Chile; cristian.martinez.s@ufrontera.cl; 8 Departamento de Kinesiología, Universidad Católica del Maule, Talca 3480112, Chile; carellano@ucm.cl

**Keywords:** inhibition, working memory, cognitive flexibility, cold and hot executive functions, mental health, physical activity, mental disorders, emotions

## Abstract

Executive functions are often impaired in individuals with schizophrenia spectrum disorders. Understanding the impact of physical exercise on these cognitive domains is essential for developing effective interventions. The aim of this review is to assess the effect of physical exercise on executive functions in adults diagnosed with schizophrenia spectrum disorders. A systematic search was conducted in Web of Science, PubMed, Scopus, and EBSCO, initially from inception through January 2024, followed by an update through January 2025. Studies involved adults diagnosed with schizophrenia spectrum disorders, employed physical exercise as an intervention, and measured executive functions as outcomes. The selection followed PRISMA guidelines, with inclusion determined by consensus among multiple reviewers. Data extraction and risk of bias assessment were conducted independently by two reviewers using the Cochrane RoB 2 tool and GRADE approach for certainty of evidence. Meta-analyses were performed using random-effects models, with effect sizes (ES) and 95% confidence intervals (CI) calculated for each outcome. From 1517 records, 9 studies were included in the meta-analysis. The analysis revealed a small but significant effect of physical exercise on working memory (ES = 0.300, 95% CI = 0.060–0.539, *p* = 0.014; *I*^2^ = 0.0%, *Q* = 2.2, *p* = 0.951) and a non-significant effect on emotion recognition (ES = 0.51, 95% CI = −0.291–1.303, *p* = 0.213; *I*^2^ = 83%), inhibition (ES = 0.156, 95% CI = −0.173 to 0.484, *p* = 0.353; *I*^2^ = 0.0%, *Q* = 1.1, *p* = 0.781), and cognitive flexibility (ES = 0.240, 95% CI = −0.270 to 0.749, 95% PI = −1.706 to 2.185; *p* = 0.356; *I*^2^ = 53.2%, *Q* = 3.0, *p* = 0.094). Physical exercise, particularly aerobic exercise, appears to have a small beneficial effect on working memory in individuals with schizophrenia spectrum disorders. However, the evidence for its effect on emotion recognition is less clear and may be influenced by the type of exercise, such as yoga. Further research is needed to provide more robust conclusions. PROSPERO registration number: CRD42023392295.

## 1. Introduction

Schizophrenia spectrum disorders are detrimental disorders associated with social and neurocognitive problems that can affect the patient’s professional and life goals [1,2,3]. In chronic schizophrenia, the range of impairments extends to a broader array of higher-order cognitive functions, including attention, problem-solving, core executive functions, and social cognition [4,5], as well as brain structural correlates of functional impairments [4]. A recent umbrella review [6] identified 63 systematic reviews on cognitive functions in schizophrenia. It revealed that individuals with schizophrenia exhibit more pronounced cognitive impairments than healthy controls and those with other affective disorders, particularly in processing speed, verbal memory, and working memory, irrespective of pharmacological treatment.

Executive functions refer to a set of cognitive abilities that enable the regulation and management of emotions, behaviors, and other mental processes in voluntary tasks [7]. They can be categorized as ‘cold’ or ‘hot’ [8]. Cold executive functions are traditionally classified into three main dimensions: working memory, inhibition, and cognitive flexibility, along with another set of high-level cognitive skills that include reasoning, problem-solving, and planning [7]. These functions are essential for tasks that require logical thinking, abstract reasoning, and attentional control, such as solving mathematical problems, following multi-step instructions, or making decisions based solely on factual information. Hot executive functions, on the other hand, refer to the processing of information related to reward, emotion, and motivation, encompassing skills such as emotional regulation and social cognition [8]. For instance, deciding whether to take a financial risk based on potential rewards, interpreting social cues, or controlling impulsive reactions in emotionally charged situations all rely on hot executive functions. Both types of executive functions are not mutually exclusive, as they depend on contextual information to define their functional nature [8]. Hot executive functions, including emotion processing and theory of mind, are impaired in schizophrenia [9,10]. Both ‘cold’ and ‘hot’ executive functions have been identified as predictors of global functional outcomes—such as self-care, occupational performance, and social functioning—in individuals with both early-onset psychosis and chronic schizophrenia [11,12]. While these two types of executive functions are often described separately, they are interdependent and shaped by contextual demands. For example, a decision making process that starts as purely cognitive (cold) may shift toward emotional involvement (hot) when personal stakes or social factors come into play.

Research indicates that individuals with schizophrenia exhibit the lowest levels of activity, averaging 37.5 min per day of moderate-to-vigorous physical activity (95% confidence interval 29.1 to 46.0 min), compared to people with other severe mental illnesses [13]. A sedentary lifestyle and low levels of physical activity among individuals with schizophrenia are associated with poorer cognitive performance [14]. Conversely, physical exercise can serve as a primary or adjunctive therapy to ameliorate clinical symptoms (including positive, negative, and cognitive symptoms), enhance quality of life, improve global functioning, and promote neuroplasticity and neurogenesis [15,16,17,18,19].

Chronic exercise interventions among individuals with schizophrenia have revealed a correlation between exercise and cognition, depending on the quantitative parameters of the exercise regimen such as frequency, intensity, and duration [16,17,19]. On the other hand, the qualitative characteristics of exercise, particularly its type, have garnered increasing attention in exercise–cognition research [20]. Previous studies have explored both the general and specific cognitive benefits of exercise in individuals with schizophrenia, investigating various modalities such as combined physical and cognitive training [21], aerobic exercise [22], and mindful exercise [23]. Furthermore, there is a growing interest in examining the role of the exercise context as a moderator of its effects on cognition and mental health [24,25].

Regarding the type of exercise, although there are studies that aim to broaden the analysis beyond aerobic exercise [26], the latter remains predominant in research focused on individuals with schizophrenia across multiple outcome domains [16,22,27,28,29,30,31], particularly cognitive [15,22,32] and socio-emotional domains [28]. However, the pattern of results from evidence syntheses is not univocal. Two meta-analyses [15,32] suggest that aerobic exercise leads to large improvements in global cognition, working memory, and attention, as well as in social cognition [15]. In contrast, another meta-analysis [33] did not find support for the beneficial effects of aerobic exercise on working memory and attention, whereas it did find positive effects on higher-level executive functions such as reasoning and problem-solving. Among the factors that may have led to inconsistent conclusions, there are differences in how meta-analyses are conducted, which pools of articles were selected, how outcomes were classified, and assessment tools combined. Indeed, it is an issue of continued debate how best to classify assessments as to the domain(s) of cognition of interest, especially when cognitive assessments are impure and may rely upon multiple cognitive domains [34]. The distinction between ‘cold’ and ‘hot’ cognition has been made with specific regard to executive function [8]. Indeed, previous reviews [15,32,33] did not analyze the effects of physical exercise on ‘cold’ and ‘hot’ executive functions, which could enhance our understanding of the impact of physical exercise on different aspects of executive functioning.

Therefore, this study aims to synthesize the existing scientific literature on the effects of physical exercise on ‘cold’ and ‘hot’ executive functions in adults diagnosed with schizophrenia spectrum disorders, comparing these effects to both active and passive control conditions. This systematic review, supplemented by meta-analysis, seeks to provide insights into the evidence regarding the impact of physical exercise on these executive functions, thereby informing therapeutic decisions aimed at mitigating the functional impairments faced by individuals with these conditions [35,36]. The research question of this study is as follows: In adults diagnosed with schizophrenia spectrum disorders, what are the effects of exercise interventions on the ‘cold’ and ‘hot’ dimensions of executive functions, as compared to active or passive control conditions?

## 2. Materials and Methods

This review and meta-analysis were guided by PRISMA guidelines [37]. The protocol showing the methodology was previously published by [38]; more detailed information can be found there.

### 2.1. Eligibility Criteria

Criteria for inclusion and exclusion were delineated utilizing the PICOS (Population, Intervention, Comparison, Outcome, Study design) categories [39] and presented in Table 1. The inclusion of non-randomized studies acknowledges that the nature of physical exercise interventions often makes randomization impractical or unethical in certain contexts [40].

### 2.2. Sources of Information

PubMed, Web of Science, EBSCO, and Scopus were used based on their extensive coverage of peer-reviewed literature and their relevance to health, exercise science, and multidisciplinary research. PubMed was included for its strong focus on biomedical and life sciences research, Web of Science for its broad citation tracking and multidisciplinary scope, EBSCO for its access to specialized health and social sciences databases, and Scopus for its comprehensive indexing of peer-reviewed literature across various disciplines. This selection ensures a diverse and high-quality pool of studies relevant to the research topic. The initial search included articles indexed from inception through January 2024. Subsequently, this search was updated through January 2025 (update date: 13 January 2025). In addition to database searches, efforts were made to identify relevant studies through secondary sources, such as references cited in review articles retrieved from these databases.

### 2.3. Search Strategy

A systematic search was conducted by two authors (F.C.-O. and N.P-R.), without restrictions on participant gender, age, other demographic characteristics, or publication date. The search process began after the protocol was accepted for publication [38]. To facilitate the search, the strategy outlined in the protocol was followed, incorporating both Medical Subject Headings (MeSH) and free-text terms related to three primary categories: executive function, physical exercise, and schizophrenia. Boolean operators (OR/AND) were used to combine the terms. For category 1, the terms included “executive function*” OR “cognitive function*” OR cognition OR “inhibitory control” OR “inhibition” OR “interference control” OR “executive control” OR “working memory” OR “updating” OR “cognitive flexibility” OR “shifting” OR “switching” OR “social cognition” OR “emotion regulation” OR “emotion recognition” OR “decision making”. Category 2 encompassed terms such as sport OR “modified sport” OR fitness OR exercise OR “physical activity” OR athletics OR “resistance training” OR “sport practice” OR “mindful movements” OR “mindfulness practice*” OR “yoga” OR “team game” OR “soccer”. For category 3, the terms used were schizoph* OR catatonia OR “schizoaffective disorder” OR “schizophreniform disorder” OR “schizoid personality disorder” OR “psychotic disorder” OR “schizophrenia spectrum and other psychotic disorders”. The search strategy is detailed in Supplementary Table S1.

### 2.4. Selection Process

A flowchart was used following the PRISMA guidelines [37]. Upon identification of documents from the databases, duplicates were eliminated using EndNote. Additionally, manual deletions were made by one author (N.P-R.). Subsequently, titles and abstracts were reviewed by two authors (F.C.-O. and N.P-R.) to assess eligibility. Furthermore, the reference lists of included articles and reviews uncovered during the search were evaluated to identify potentially suitable studies. In instances of discordance between the authors’ decisions, consensus was reached with the involvement of a third author (C.C.-J.).

### 2.5. Data Extraction and Management

For each study, the following data were identified: year of publication, author, sample size, characteristics of participants (sex, age, fitness level, psychiatric diagnosis and severity, comorbidities, pharmacological treatment, and social-related information, e.g., level of social support, family structure, and academic level]), description of the physical exercise program, weekly frequency of the intervention, duration of the intervention (in weeks), duration (in minutes) and intensity of sessions and/or exercises (e.g., heart rate; Borg scale), dimensions of ‘cold’ executive function assessed (working memory, inhibition, cognitive flexibility) and ‘hot’ social cognition (emotion recognition, Theory of Mind, social perception, and social cognition), tasks or test used for assessment, and description of the control condition. One author (F.C.-O.) conducted the data extraction process, which was subsequently reviewed by a second author (N.P.-R.). Moreover, discrepancies were solved through consensus with a third author (R.R.-C.). In cases where the data were not clearly or fully provided in the document, attempts were made to contact the authors of the respective studies. Authors were contacted a maximum of two times within a two-week period. Studies with unresolved data issues or unattainable data were excluded from the analysis.

### 2.6. Risk of Bias Assessment and Certainty Assessment

RoB 2 was used for the assessment of bias risk in randomized controlled trials [41]. The ROBINS-I tool was used to assess the three non-randomized studies [42]. The evaluation of bias risk was performed independently by two authors (F.C.-O. and C.C.-J.), with any discrepancies resolved through consultation with a third author (N.P.-R.). All studies meeting the inclusion criteria were considered for analyses, regardless of the RoB 2 or ROBINS-I results. Risk of bias refers to the potential systematic errors in the design or conduct of a study that may affect the validity of its results. The risk of bias is assessed as low, high, or uncertain for each individual item, as well as an overall assessment for the study.

In contrast, the certainty of evidence evaluates the overall confidence in the body of evidence regarding a specific outcome. This assessment considers factors such as study design, risk of bias, inconsistency, indirectness, imprecision, and publication bias. To synthesize and assess the certainty of evidence for each outcome, the GRADE (Grading of Recommendations, Assessment, Development, and Evaluation) method was utilized [43]. The evidence was categorized as high, moderate, low, or very low certainty [44,45].

### 2.7. Meta-Analysis

The meta-analysis included emotion recognition, working memory, inhibition, and cognitive flexibility, as these were the only outcome measures for which at least three studies provided the necessary data for the analysis [46,47]. Effect sizes (ES) were calculated using Hedges’ g with a 95% confidence interval (CI) and prediction interval. The ES estimation relied on pre- and post-intervention mean and standard deviation values in experimental versus control groups. The DerSimonian and Laird random effects model was used for meta-analyses. The ES was categorized as follows: <0.2 trivial, 0.2–0.6 small, >0.6–1.2 moderate, >1.2–2.0 large, >2.0–4.0 very large, and >4.0 extremely large. Heterogeneity was assessed using the *I*^2^ statistic, with values indicating low, moderate, and high levels of heterogeneity. Comprehensive Meta-Analysis software (version 2) facilitated all analyses, with statistical significance set at *p* ≤ 0.05.

## 3. Results

### 3.1. Study Selection

**Figure 1:**
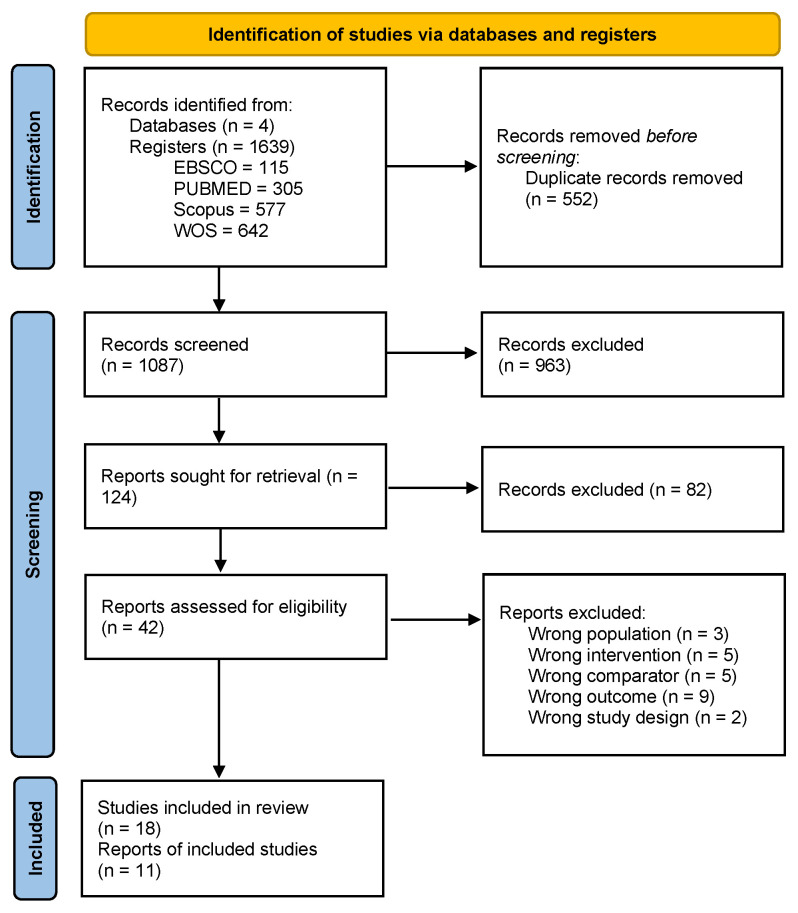
Flow diagram from PRISMA guidelines.

The process of searching and selecting studies has been depicted in the flowchart suggested by PRISMA (Figure 1).

As shown in Figure 1, 1639 studies were found in the four databases used (115 in EBSCO, 305 in PubMed, 577 in Scopus, and 642 in WoS). First, duplicate articles were eliminated with the EndNote package (n = 231); additionally, the rest of the articles were reviewed for duplicates (n = 321), thus eliminating a total of 552 articles. After this, the titles and abstracts of the remaining studies (n = 1087) were read, excluding 1045 articles that did not meet all the inclusion criteria.

Finally, 42 studies were fully read and evaluated according to the inclusion and exclusion criteria. Of these studies, three were removed because they were carried out including people with other disorders alongside people with schizophrenia in the same sample without separating them by specific diagnosis (two studies with bipolar and the remaining one with depression). Then, five studies were removed because they did not involve physical exercise interventions with a minimum duration of 4 weeks. Moreover, five studies were excluded due to the comparator: three because they included several groups, all considered experimental, and another two because they did not allow to identify the distinguishing features of the experimental and the control group (i.e., the group indicated by authors as the active control group performed brisk walking whose intensity could be too similar to that of the experimental group assigned to structured physical exercise). Indeed, other authors considered this type of activity an intervention [48]. A further nine studies were eliminated because they did not evaluate any executive function or only showed the overall score and not by component (e.g., the Mini-Mental State Examination of global cognition). Lastly, two studies were excluded because they did not employ a longitudinal design. The “Supplementary S2” file includes an Excel sheet with a detailed list of all the studies read in full.

Two articles lacked data for meta-analysis, which were not obtained upon request to their respective authors. Therefore, 18 articles were included in the qualitative synthesis (Table 2), and 11 articles were included in the meta-analysis (three for emotion recognition, eight for working memory, three for inhibition, and four for cognitive flexibility). Table 2 presents data on participants’ characteristics such as initial cognitive status, severity of the diagnosis, some psychosocial variables, and associated comorbidities.

### 3.2. Characteristics of Studies

Interventions consisted of aerobic exercises (e.g., cycle ergometer, dancing, and walking) (n = 14), also combined with cognitive training (n = 2), or yoga sessions (n = 5). Most of the control groups were passive controls that followed the usual treatment (n = 7), followed by waitlist (n = 3); physically active controls that performed stretching and flexibility exercises (n = 4); or non-physically active controls that performed cognitive exercises such as coloring, occupational therapy, or healthy conversations (n = 4). The duration of the interventions ranged from 1 to 4 months, from two to six sessions per week, and with sessions being 20–60 min throughout the different studies. These and other data related to the intensity and other treatments are shown in Table 3.

Lastly, studies evaluated ‘cold’ executive functions, with 12 studies assessing working memory, eight assessing cognitive flexibility, and four assessing inhibition; and hot executive functions, with seven studies assessing emotion recognition, three the broader construct of social cognition, one theory of mind, and one social perception). Table 3 shows the instruments used in this review for assessing executive functions, according to the distinction between ‘cold’ and ‘hot’ dimensions.

### 3.3. Risk of Bias

**Figure 2:**
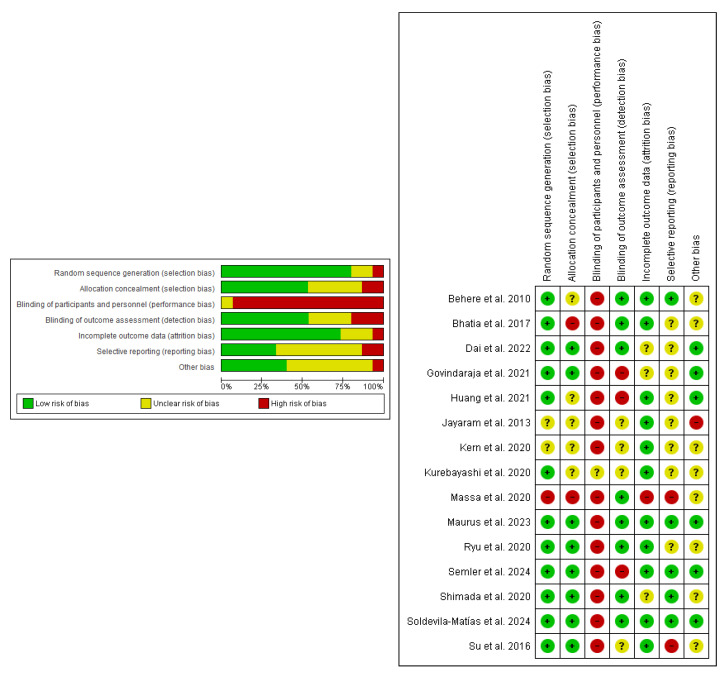
Risk of bias summary for RCT studies: review authors’ judgments about each risk of bias item for each included study. Green, yellow, and red colors: low, uncertain, and high risk of bias, respectively. Behere et al. (2010) [49], Bhatia et al. (2017) [51], Dai et al. (2022) [53], Govindaraj et al. (2021) [54], Huang et al. (2021) [55], Jayaram et al. (2013) [56], Kern et al. (2020) [48], Kurebayashi et al. (2022) [57], Massa et al. (2020) [58], Maurus et al. (2023) [59], Ryu et al. (2020) [61], Semler et al. (2024) [64], Shimada et al. (2020) [62], Soldevila-Matías et al. (2024) [65] and Su et al. (2016) [63].

Risk of bias was assessed using the RoB 2 tool in randomized controlled trials [41]. Figure 2 shows that 95% of the studies (n = 13) presented a high risk of performance bias (item 3). The rest of the items presented a 20 to 5% risk of bias. Figure 2 also shows the specific data for each study; four of them present the highest risk of bias. Overall risk of bias was assessed according to the worst-case scenario; all studies were rated as high risk of bias except one, which was rated as uncertain [57].

**Figure 3:**
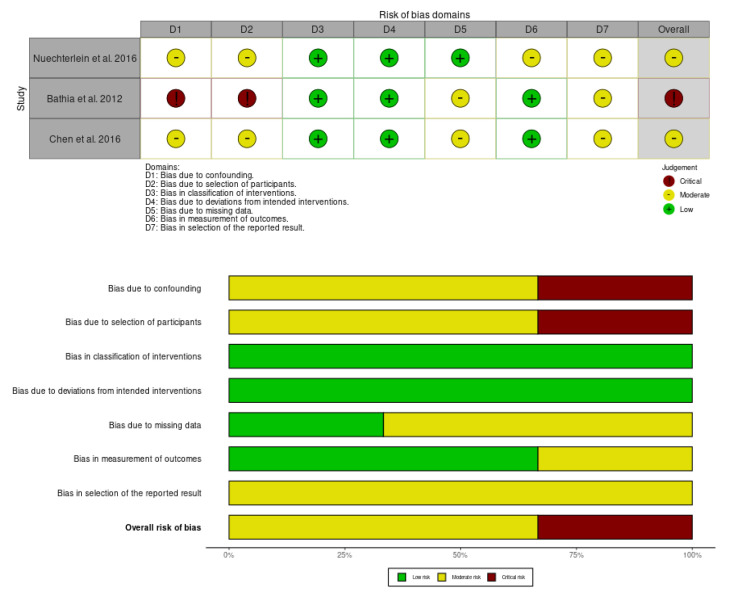
Risk of bias summary for non-RCT studies: review authors’ judgments about each risk of bias item for each included study. Low risk: green, uncertain risk: yellow, high risk: red. Nuechterlein et al. (2016) [60], Bathia et al. (2012) [50] and Chen et al. (2016) [52].

The risk-of-bias assessment for non-randomized studies was conducted using the ROBINS-I tool [42]. Figure 3 shows that two of the three studies exhibit a moderate overall risk of bias, while the remaining study shows a high risk of bias. Bias due to confounding and due to selection of participants shows 20% risk of bias.

### 3.4. Meta-Analysis Results for Emotion Recognition

**Figure 4:**
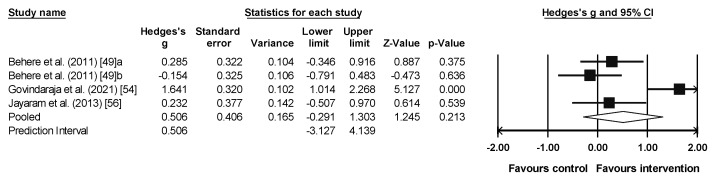
Forest plot of emotion recognition results [49,54,56]. Note: a = yoga group, b = exercise group. The squares represent the effect size (Hedges’ g) for each study, with the size of the square proportional to the weight of the study in the meta-analysis. The horizontal lines through each square indicate the 95% confidence intervals (CI) for the individual studies. The diamond at the bottom represents the pooled effect size, with its width reflecting the 95% CI. The vertical line at 0.0 represents the line of no effect. Values to the left of the line favor the control group, while values to the right favor the intervention. The arrows indicate that the confidence interval extends beyond the scale shown on the plot.

The meta-analysis results for emotion recognition included three studies with four experimental groups (n = 106), and three control groups (n = 63). A small and non-significant effect and a high heterogeneity was observed for both the experimental and the control groups (ES = 0.51, 95% CI = −0.291–1.303, PI = −3.127–4.139, *p* = 0.213; *I*^2^ = 83%, Q = 17.7, *p* = 0.001; Figure 4). Only the study by Govindaraj et al. (2021) [54] was significant, being the one with the largest effect size (Figure 4).

### 3.5. Meta-Analysis Results for Working Memory

**Figure 5:**
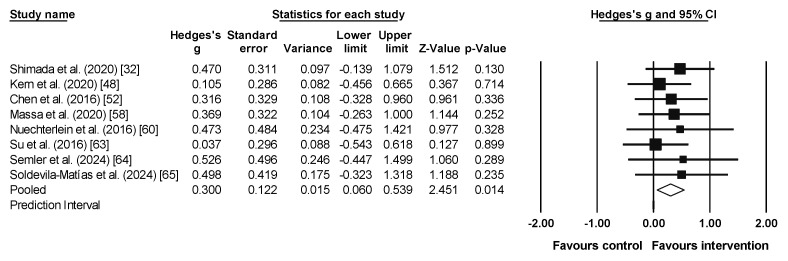
Forest plot of working memory results [32,48,52,58,60,63,64,65]. The squares represent the effect size (Hedges’ g) for each study, with the size of the square proportional to the weight of the study in the meta-analysis. The horizontal lines through each square indicate the 95% confidence intervals (CI) for the individual studies. The diamond at the bottom represents the pooled effect size, with its width reflecting the 95% CI. The vertical line at 0.0 represents the line of no effect. Values to the left of the line favor the control group, while values to the right favor the intervention. The arrows indicate that the confidence interval extends beyond the scale shown on the plot.

Eight studies provided eight experimental groups (n = 135) and eight control groups (n = 114). A small significant effect (with small heterogeneity) favored the intervention group (ES = 0.300, 95% CI = 0.060–0.539, *p* = 0.014; *I*^2^ = 0.0%, Q = 2.2, *p* = 0.951; Figure 5). When the studies of Chen et al. [52] and Nuecherltein et al. [60] (i.e., non-RCTs) were excluded from the meta-analysis, the pooled ES was 0.28 (95%CI 0.02 to 0.55, *p* = 0.039, *I*^2^ = 0.0%).

### 3.6. Meta-Analysis Results for Inhibition

**Figure 6:**
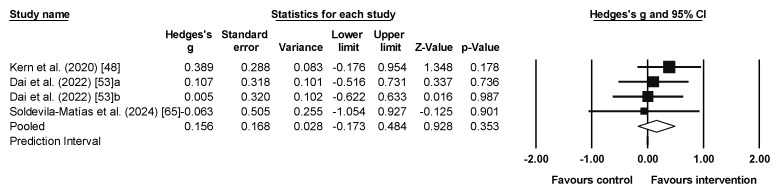
Forest plot of inhibition results [48,53,65]. The squares represent the effect size (Hedges’ g) for each study, with the size of the square proportional to the weight of the study in the meta-analysis. The horizontal lines through each square indicate the 95% confidence intervals (CI) for the individual studies. The diamond at the bottom represents the pooled effect size, with its width reflecting the 95% CI. The vertical line at 0.0 represents the line of no effect. Values to the left of the line favor the control group, while values to the right favor the intervention. The arrows indicate that the confidence interval extends beyond the scale shown on the plot. The letters “a” and “b” distinguish between different groups that share the same study.

Three studies provided four experimental groups (n = 98) and three control groups (n = 85). A trivial and non-significant effect (with small heterogeneity) was observed (ES = 0.156, 95% CI = −0.173 to 0.484, *p* = 0.353; *I*^2^ = 0.0%, *Q* = 1.1, *p* = 0.781; Figure 6).

### 3.7. Meta-Analysis Results for Cognitive Flexibility

**Figure 7:**
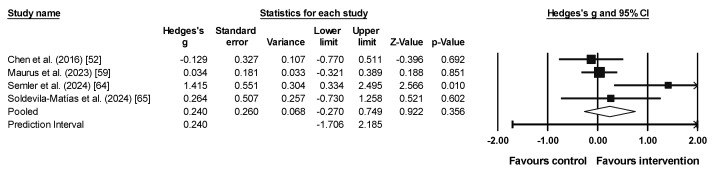
Forest plot of cognitive flexibility results [52,59,64,65]. The squares represent the effect size (Hedges’ g) for each study, with the size of the square proportional to the weight of the study in the meta-analysis. The horizontal lines through each square indicate the 95% confidence intervals (CI) for the individual studies. The diamond at the bottom represents the pooled effect size, with its width reflecting the 95% CI. The vertical line at 0.0 represents the line of no effect. Values to the left of the line favor the control group, while values to the right favor the intervention. The arrows indicate that the confidence interval extends beyond the scale shown on the plot.

Four studies provided four experimental groups (n = 67) and four control groups (n = 61). A small non-significant effect (with moderate [near significant] heterogeneity) was observed (ES = 0.240, 95% CI = −0.270 to 0.749, 95% PI = −1.706 to 2.185; *p* = 0.356; *I*^2^ = 53.2%, *Q* = 3.0, *p* = 0.094; Figure 7).

### 3.8. Certainty of the Evidence

Emotion recognition, working memory, inhibition, and cognitive flexibility variables were evaluated with high certainty and moderate certainty, respectively (Table 4).

## 4. Discussion

The aim of this systematic review, complemented by meta-analysis, was to synthesize the existing scientific literature on the impact of physical exercise on executive function in adults diagnosed with schizophrenia spectrum disorders, in comparison to both active and passive control conditions.

### 4.1. Non-Significant Results: Emotion Recognition, Inhibition, and Cognitive Flexibility

Facial emotion recognition is a component of social cognition, referring to the ability to detect emotional patterns. Individuals with schizophrenia usually present an impairment in this ability [49,54,56]. All three studies that assessed emotional recognition in this review were RCTs and used the same assessment instrument (TRENDS). Our meta-analytical results did not show any significant effect of physical exercise interventions on emotion recognition. As depicted in results, all three analyzed studies indicated favorable outcomes for the experimental group, including the yoga versus control comparison in Behere et al. [49]. Nevertheless, one of the two comparisons within the study [49], exercise versus control, demonstrated superior performance changes over time for the control group. Notably, yoga was the intervention used in the three studies reporting results in favor of the intervention, with the largest effect size reported [54]. Among the assessed variables that could help explain this largest efficacy, it is useful to note that the Govindaraj groups [54] had the lowest dropout rate (see Table 3). On the other hand, a thorough comparison of quantitative exercise parameters that could help explain differences in effect size was not possible, as data on frequency, intensity, or duration were not fully detailed in all three studies. Additionally, several moderating factors could have influenced the results, including individual differences in baseline emotional processing abilities, such as facial recognition and emotional regulation, particularly difficulties in downregulating responses to negative stimuli and filtering distractors in schizophrenia [66]; specific characteristics of the exercise interventions, such as intensity, duration, type, and adherence [28,67,68]; and participants’ overall mental state, including symptom severity, stage of illness, and antipsychotic medication [28]. These moderators could explain the variability in the therapeutic response observed across participants. However, more research is still needed in this area to provide more accurate answers.

It might be relevant for future research to fully detail these aspects to really assess all the characteristics of the intervention, as they might influence the underlying physiological mechanisms [69].

Previous studies found that oxytocin could be related to emotional competence [70,71]. Jayaram et al. [56] supported the role of adjunctive yoga therapy in managing schizophrenia and showed an increase in endogenous plasma oxytocin levels in schizophrenia patients undergoing yoga therapy. Therefore, this could be a possible mechanism linking the practice of yoga with the improvement of emotional aspects. Furthermore, studies such as those by Domes et al. (2007) and Marsh et al. (2010) [70,71] have reported enhancements in the mirror neuron system, a network of visuo-motor neurons that are activated both by performing an action and by observing the same action in others [72]. This system seems to be responsive to other people’s intentional actions, facilitating the understanding of emotions, fostering empathy, social connection, and the ability to interpret and respond to emotional signals [73,74]. However, it is important to note that a high degree of heterogeneity exists, which warrants caution in interpreting the results. This heterogeneity may stem from variations in the interventions assessed across the included studies, such as differences in their nature, dosage, or duration. However, due to the limited number of available studies, we are unable to confirm this hypothesis, which also restricts the ability to conduct more detailed analyses, such as subgroup analyses or meta-regressions, to identify sources of variability. Furthermore, with such a small sample of studies, the reliability of heterogeneity metrics may be compromised, complicating the interpretation of the findings.

Cognitive flexibility enables individuals to adapt to new circumstances and adjust strategies according to environmental demands [7]. It is a key function in which individuals with schizophrenia exhibit difficulties, limiting their cognitive adaptation capacity. The results obtained in this dimension show a small, non-significant effect; however, the study by Semler et al. [64] deviates from the others, presenting a clear favorable outcome for the intervention group. The discrepancy in the findings may be attributed to methodological differences between studies, such as sample heterogeneity or the assessment tools used (in this case, a difference might be observed between the use of the Wisconsin Card Sorting Test and Trail Making Test across the included studies). On the other hand, inhibition, defined as the ability to suppress automatic or irrelevant responses [7], consistently showed a small, non-significant effect. Once again, methodological aspects could influence the results, but it is also essential to consider the characteristics of the interventions provided, which may not have offered the level of stimulation necessary to generate improvements in cognitive performance, thus, affecting the results [60]. In this regard, the intensity of the exercise performed could be a relevant factor to consider [27,67].

### 4.2. Significant Results: Working Memory

For this variable, the meta-analysis reported a small but significant effect in favor of intervention groups versus control groups, including cognitive work [52,60], stretching [48,58,63], and treatment as usual (TAU) [62]. Additionally, consistency analysis performed by GRADE yielded high-certainty data for the meta-analysis.

These results are consistent with previous meta-analyses by Firth et al. (2017) and Shimada et al. (2022) [15,32], who noted improvements in working memory. Just like these previous studies [15,32], our results were obtained through aerobic exercise interventions; only one of them combined aerobic exercises with cognitive activities [60], but did not show any difference between the intervention and control group. However, it should be noted that these results do not prove that other types of physical exercise do not show beneficial results, as no studies showing results from other activities have been reported. Similar results have been found in previous meta-analyses for other diseases like depression [75] or in older populations with healthy cognition [76]. Neurophysiological reasons that would explain these results have been proposed, as improvements in neuronal plasticity, neurotrophic factors, neurogenesis, hippocampal structure, or the brain-derived neurotrophic [77,78]. In this same line, it has been suggested that exercise benefits cognition through mechanisms operating at different levels (cellular/molecular, brain structure and function, psychosocial), with variations depending on age, baseline health conditions, brain regions, or cognitive abilities analyzed, and the parameters of exercise used in the interventions [79]. Specifically in schizophrenia, although current evidence remains contradictory regarding the mechanisms underlying cognitive improvements following physical exercise interventions [27], there are precedents supporting the importance of neurogenesis [80,81] and increases in brain-derived neurotrophic factor (BDNF) [82] as potential mechanisms in this special population.

### 4.3. Limitations

Although the results of this review suggest that exercise may be beneficial for working memory, it is important to note that there remains a lack of scientific literature examining each of the skills related to executive functions in isolation. Therefore, these findings should be interpreted with caution. Several studies assessed executive functions in a general manner or did not provide specific data for each variable, including inhibition and cognitive flexibility, which also did not yield significant results. As a consequence, those studies could not be included in the meta-analysis. Additionally, publication bias must be considered, as studies with unfavorable results may not have been published. Similarly, the absence of reported adverse effects in the included studies does not necessarily imply their nonexistence; it may simply reflect that such effects were not assessed, potentially due to an assumption that the intervention would not lead to them.

Another potential limitation lies in the limited number of studies that reported all the required results, as stipulated by the methodology, to be included in both the meta-analysis and the moderator analysis. Furthermore, not all included studies were able to fully implement randomization of their samples due to their inherent design, which may have introduced a potential bias that could not be controlled. The considerable diversity in the instruments used to assess executive function could also be considered a limitation, contributing to the increased heterogeneity in the obtained results, reflecting the lack of consensus among authors on this matter. Another limitation is that, although a moderator analysis can technically be conducted even with a single study in a category, the reliability of such analyses is considerably low. As a result, the limited data available for certain exercise prescription variables (e.g., intensity, frequency) restricts our ability to draw definitive conclusions regarding their moderating effects.

Based on these limitations, researchers are encouraged to conduct more studies in this field, providing a detailed account of the various aspects involved in the implementation of their physical exercise interventions. This includes explicitly reporting the assessment and presence (if any) of adverse effects, which is crucial, particularly when the samples involve clinical populations. Lastly, it is suggested that future studies consider using executive function assessment tools that have strong support in the existing literature, to unify criteria and facilitate the subsequent comparison of results.

## 5. Conclusions

According to the World Health Organization [83], schizophrenia is associated with significant stigma and challenges in accessing psychological care. Therefore, exploring alternative therapeutic options, such as exercise programs, could improve the availability of affordable treatments. Additionally, exercise may help mitigate the side effects of medications, promote overall health, and reduce mortality rates among individuals with schizophrenia [16]. However, in evaluating its impact on cognitive functions, the scientific community stresses the need for further research due to inconsistent findings across studies [15,23,32,84].

This study reinforces the previously established notion that aerobic exercise can enhance working memory in individuals with schizophrenia, reporting a small yet statistically significant effect in favor of the intervention. However, no significant effects were observed on “hot” executive functions, specifically in facial emotion recognition, inhibition, or cognitive flexibility. Furthermore, the data for these variables were insufficient for meta-analysis. While this study contributes novel insights through the separate analysis of each executive function rather than presenting aggregate results, further research is necessary to draw more definitive conclusions and establish a potential dose–response relationship.

## Figures and Tables

**Table 1:** Eligibility criteria.

	Inclusion	Exclusion
Population	Adult individuals (age, 18–60 years) diagnosed with schizophrenia spectrum according to the Diagnostic and Statistical Manual of Mental Disorders (DSM), the International Classification of Diseases (ICD), or its equivalents from any version.	Major psychiatric disorders (e.g., bipolar disorder, dementia) and intellectual disabilities as comorbid conditions. Medical conditions that restrict physical activity (e.g., cardiopulmonary disease).
Intervention	Interventions involving physical exercise without restrictions regarding the type of exercise (e.g., multicomponent, aerobics, sports programs, yoga) that may include (or not) cognitive exercises.	Interventions with a duration of less than four weeks.
Comparator	Active (e.g., relaxation techniques) or passive (e.g., waiting list) control groups not exposed to physical exercise intervention.	Comparator group(s) with meaningfully different characteristic(s) other than the physical exercise intervention (e.g., sex).
Outcomes	Pre- and post-intervention data from at least one instrument for the direct assessment of ‘cold’ (working memory, inhibition, cognitive flexibility) or ‘hot’ (emotion regulation, decision making, and dimensions of social cognition) executive functions evaluated with instruments that have been previously validated (e.g., Trail Making Test, Flanker task, N-back task, or Iowa Gambling Task).	Indirect evaluation measures of the executive function, self-report questionnaires.
Study design	Randomized and non-randomized controlled studies.	Single-group studies; Case study (e.g., n < 4).

**Table 2:** Subjects’ characteristics from the included studies.

Reference	N	Sex (M/F)	Age (Years)	Diagnosis (Diagnostic Tool)	Baseline Schizophrenia Symptom Severity	Baseline Cognitive Status	Psychosocial Variables	Comorbidity
[49]	91, EG1: 34, EG2: 31, CG: 26	EG1: 18/9 EG2: 14/3 CG: 15/7	EG1: 31.3 ± 9.3 EG2: 30.2 ± 8.0 CG: 33.6 ± 9.9	Sch. (DSM-IV)	Positive symptoms: EG1: 15.1 ± 11.7, EG2: 14.9 ± 4.3, CG: 14.7 ± 6.3 Negative symptoms: EG1: 17.8 ± 4.9, EG2: 14.8 ± 3.9, CG: 14.3 ± 3.7	N. R.	N. R.	N. R.
[50]	88, EG: 65, CG: 23	EG: 43/22, CG: 11/12	EG: 31.50 ± 9.82, CG: 31.38 ± 11.14	Psychoses (DIGS and FIGS)	Global Assessment of Functioning (GAF): EG: 34.33 ± 12.78, CG: 27.22 ± 6.266	N. R.	Education in years: EG: 33.27 ± 9.815; CG: 8.79 ± 5.150 Marital status (ever married/never married): EG: 51.8%/48.2%; CG: 55.3%/44.7% Occupation [Employed/ (Unemployed or Retired)/Housewife/Student]: EG: 20%/59%/15%/6%; CG: 25%/56%/13%/6%	N. R.
[51]	286, EG1: 104, EG2: 90, CG: 92	EG1: 62/42, EG2: 62/28, CG: 57/35	EG1: 34.76 ± 9.56, EG2: 35.20 ± 9.49, CG: 35.72 ± 10.06	Sch. (DSM-IV)	SAPS: EG1: 12.44 ± 11.50, EG2: 13.30 ± 12.93, EG2: 10.52 ± 12.55 SANS: EG1: 23.58, EG2: 24.02 ± 18.10, CG: 24.82 ± 18.69	MMSE: EG1: 26.21 ± 6.01, EG2: 25.91 ± 7.24, CG: 25.05 ± 7.25	Married/not married: EG1: 51/53; EG2: 51/39; CG: 55/37. Occupation (professional specialty/ technical, sales and administrative support/ service (household, protective)/ all other occupations: EG1: 2/3/9/90; EG2: 0/1/7/82; CG: 0/3/6/83. Education (years): EG1: 10.24 ± 4.05; EG2: 9.38 ± 4.07; CG: 8.76 ± 4.54	N. R.
[52]	41; EG: 21, CG: 20	EG: 11/10, CG: 8/12	EG: 37.0 ± 9.7, CG: 36.1 ± 8.1	Sch. (DSM-IV)	Illness length (years): EG: 11.2 ± 6.3, CG: 14.7 ± 7.9 Not evaluated with severity instruments	MMSE: EG: 27.3 ± 1.8, CG: 27.4 ± 1.7	Education: high school EG: 57.1%, CG: 60.0%; College or above EG: 42.9%; CG: 40.0% Residence: with families EG: 90.5%, CG: 100%; alone EG: 9.5%, CG: 0% Marital status: single EG: 85.7%, CG: 85%; Married EG: 0%, CG: 10%; Divorced EG: 14.3%, CG: 5%	N. R.
[53]	82, EG1: 25, EG2: 26, CG: 31	EG1: 20/5, EG2: 22/4, CG: 20/11	EG1: 41.40 ± 7.86, EG2: 41.50 ± 8.72, CG: 44.06 ± 8.40	Sch. (DSM-V)	PANSS general: EG1: 35.96 ± 5.30, EG2: 34.23 ± 6.26, CG: 33.87 ± 4.65	MoCA: EG1: 20.44 ± 2.02, EG2: 20.08 ± 1.98, CG: 19.97 ± 1.54	Years of education: CG: 9.16 ± 2.72; EG1: 10.00 ± 2.74; EG2: 9.00 ± 3.16	N. R.
[54]	51, EG: 26; CG: 25	EG: 15/11; CG: 19/06	EG: 33.62 ± 7.22; CG: 32.92 ± 6.40	Sch. (DSM-V)	CGI: EG: 4.62 ± 0.85, CG: 4.76 ± 1.01 SANS: EG: 47.76 ± 14.32, CG: 43.56 ± 14.72 SAPS: EG: 29.00 ± 12.87, CG: 24.72 ± 14.52	N. R.	Years of education: EG: 12.54 (3.14); CG: 11.20 ± 4.13 Married: Single = EG: 8:18; CG: 10:15	N. R.
[55]	77, EG: 33, CG: 34	EG: 15/18, CG: 13/21	EG: 41.0 ± 10.3, CG: 42.5 ± 8.7	Sch. (DSM-V)	PANSS: EG: 80.8 ± 12.6, CG: 76.6 ± 12.9	N. R.	Education, years: EG: 13.3 ± 2.2; CG: 13.3 ± 1.8 Married: EG: 11.8%; CG: 9.1 Employed: EG: 26.5%; CG: 24.2%	N. R.
[56]	27, EG: 15, CG: 12	EG: 12/3, CG: 7/5	EG: 28.33 ± 4.7, CG: 29.5 ± 8.2	Sch. (DSM-IV)	SAPS: EG: 7.8 ± 2.8, CG: 6.6 ± 2.3 SANS: EG: 13.8 ± 5.2, CG: 10 ± 4.6	N. R.	Years of education: EG: 12.54 ± 3.14; CG: 11.20 ± 4.13	N. R.
[48]	53, EG: 35, CG: 18	EG: 94% male, EC: 18/0	EG: 56.3 ± 6.2, GC: 55.7 ± 7.1	Sch. or schizoaffective disorder (DSM-V)	BPRS total: EG: 39.2 ± 11.3, CG: 45.7 ± 14.2	N. R.	Education (years): EG: 12.8 ± 1.6; CG: 12.8 ± 1.2	N. R.
[57]	18, EG: 4, CG:14	Not reported	EG: 59.7 ± 13.0, CG: 50.3 ± 14.0	Sch. (DSM-V)	PANSS: EG: 75.5 ± 13.1, CG: 71.0 ± 18.3	Neurocognitive index: EG: 54.6 ± 21.6, CG: 44.5 ± 37.7	N. R.	N. R.
[58]	38, EG: 21, CG: 17	EG: 16/5, CG: 15/2	EG: 52.52 ± 6.66, CG: 53.18 ± 10.27	Sch. (Not reported)	PANSS: EG: 62.38 ± 12.79, CG: 59.94 ± 8.99	MATRICS: EG: 26.10 ± 12.48, CG: 25.47 ± 9.87	N. R.	N. R.
[59]	180, EG: 89, CG: 91	EG: 53/36, CG: 50/41	EG: 36.84 ± 11.83, CG: 47.90 ± 12.15	Sch. (DSM-IV)	PANSS: 50.26 ± SD 11.81	N. R.	Education years: EG: 14.23 ± 3.54; CG: 14.34 ± 3.94	N. R.
[60]	16, EG: 7, CG: 9	EG: 4/3, CG: 8/1	EG: 21.8 ± 3.8, CG: 23.5 ± 5.4	Schizophrenia, schizoaffective disorder, depressed type, or schizophreniform disorder (DSM-IV)	24-item BPRS: EG: 37.0 ± 8.9, CG: 44.4 ± 11.1	Not reported	Married: Single: EG: 8:18; CG: 10:15	Not reported.
[61]	60, EG: 30, CG: 30	EG: 15/15, CG: 17/13	EG: 38.65 ± 10.11, CG: 39.00 ± 8.62	Schizophrenia or schizoaffective disorder (DSM-IV-TR)	BPRS: EG: 48.08 ± 15.64, CG: 43.75 ± 10.61	WCST (CR): EG: 51.36 ± 17.56, CG: 56.77 ± 16.56	Years of education: EG: 13.35 ± 3.38; CG: 14.63 ± 2.123	BDI: EG: 22.12 ± 10.02, CG: 20.71 ± 8.14 STAI: EG: 47.23 ± 8.02, CG:45.29 ± 10.17
[62]	41, EG: 20, CG: 21	EG: 9/11, CG: 9/12	EG: 21.62 ± 3.63; CG: 18.75 ± 3.89	Schizophrenia: EG: 16, CG: 4; schizoaffective disorder: EG: 18, CG: 3 (DSM-V)	PANSS: EG: 112.65 ± 19.43, CG: 105.75 ± 11.33	Not reported	Education (year): EG: 12.57 ± 2.04; CG: 11.95 ± 1.61 Employment experience (% yes): EG: 76.19%; CG: 80.00%	Not reported
[63]	44, EG: 22, CG: 22	EG: 10/12, CG: 10/12	EG: 37.64 ± 8.23, CG: 36.68 ± 8.33	Schizophrenia or schizoaffective disorder (DSM-IV)	PANSS positive symptoms: EG: 16.05 ± 6.48, 18.18 ± 7.11, PANSS negative symptoms: EG: 22.36 ± 9.65, CG: 21.82 ± 8.72	WAIS-III total IQ: EG: 87.83 ± 14.06, 82.11 ± 8.36	Years of education: EG: 12.55 ± 2.86; CG: 11.50 ± 2.37	Not reported
[64]	15, EG: 8, CG: 7	EG1: 5/3CG: 7/0	EG: 35.00 ± 9.87 CG: 29.86 ± 9.14	Paranoid schizophrenia (ICD-10)	PANSS: EG: 72.88 ± 22.78, CG: 82.14 ± 7.31	N. R.	N. R.	N. R.
[65]	17, EG: 12, CG: 5	N. R.	N. R.	Schizophrenia (DSM-V)	N. R.	N. R.	N. R.	N. R.

BDI = Beck’s Depression Inventory; BPRS = Brief Psychiatric Rating Scale; CG = control group; CGI = Computer-Generated Imagery; DIGS = Hindi version of the Diagnostic Interview for Genetic Studies; DSM = Diagnostic and Statistical Manual of Mental Disorders; EG = experimental group; FIGS = Family Interview for Genetic Studies; IQ = Intelligence Quotient; MATRICS = MATRICS Consensus Cognitive Battery; MMSE = Mini-Mental State Examination; MoCA = Montreal Cognitive Assessment; N. R. = Not Reported; PANSS = Positive and Negative Syndrome Scale; SANS = Scale for Assessment of Negative Symptoms; SAPS = Scale for Assessment of Positive Symptoms; Sch. = Schizophrenia; STAI: State-Trait Anxiety Inventory; WAIS = Wechsler Adult Intelligence Scale. ICD = International Classification of Diseases.

**Table 3:** Intervention characteristics of the included studies.

Reference/Design	Exercise Program	Control Condition	Compliance with the Intervention Program	Intervention Length	Frequency	Session Length	Intensity	Other Treatments	EF Dimension and Instrument
[49] RCT	Yoga (EG1): SVYASA, a program combining loosening, breathing exercises, various postures (sitting, supine, prone), and relaxation techniques (excluding meditation). The first 2 months guided by instructors; for the next 2 months, the patient’s caregivers were instructed to monitor the yoga therapy at home. Exercise group (EG2): adapted National Fitness Corps program, focusing on brisk walking, jogging, and postures in various positions (standing, sitting) with relaxation.	Waitlist	Drop-outs: 7 in yoga group, 14 in exercise group, and 4 in waitlist group. Final sample: 27 in yoga group, 17 in exercise group, and 22 in waitlist group.	4 months	Not reported	60 min	Not reported.	Antipsychotic (CPZ equivalents in mg/day): EG1: 335.0 ± 205.3 [400], EG2: 297.9 ± 150.9 [300], CG: 340.0 ± 172.4 [675]	ER: TRENDS
[50] Non-RCT	Yoga: supervised by a professor, combined postures (Asanas) and breathing exercises (Pranayama). Sessions started with chanting and warm-up, followed by diverse poses (standing, lying, prone, and sitting) designed to synchronize movement with breath. Saturdays featured sinus-cleansing practices (Kriya/Jalneti) for added benefits.	TAU	89 participants drop out in different moments of yoga intervention.	21 days	6/week (no Sundays)	60 min	Not reported.	All participants received conventional pharmacological and non-pharmacological treatment from their psychiatrists and therapists throughout the study.	CF: CNB in a web base interface WM: CNB in a web base interface similar to N-back ER: CNB in a web base interface
[51] RCT	Yoga (EG1): comprised postures, breathing and weekly nasal cleansing, by qualified instructors to groups of participants (1–5). Physical exercise (EG2): brisk walks, followed by jogging and directed aerobic exercises. The participants were advised to continue TAU, YT, or PE past the training period. By qualified instructors to groups of participants (1–5).	TAU	Dropout (n = 48) in the YT and PE groups: transportation difficulties (YT 2; PE 3; TAU 3), staying far away (YT 3; PE 2); not able to arise early (YT 5); symptoms aggravated (YT 2; PE 2); being transferred out of Delhi (YT 3; PE 1; TAU 3)	21 days	6/week (no Sundays, no public holidays)	60 min	Not reported.	Not reported.	CF: CNB in a web base interface WM: CNB in a web base interface ER: CNB in a web base interface
[52] Non-RCT	Aerobic dance intervention: 5 min warm-up, 40 min of aerobic dance with a 5 min break and 10 min cool down. Simple movements choreographed by an occupational therapist. Motivational strategies were implemented.	Coloring and handwriting activities (text copies of 300 words) and attentional control.	In EG (n = 1) and in CG (n = 3), participants withdrew from the program because of unstable psychiatric symptoms. Average attendance EG = 73% and CG = 71%.	3 months (38 sessions)	3/week	50–60 min	52.8% HRR	No changes in antipsychotic medications between the previous 3 months	CF: TMT-B WM: Semantic Association of Verbal Fluency Test
[53] RCT	Aerobic exercise training (EG1): aerobic exercise by qualified instructors, music warm-up exercise (10 min) and walking on a treadmill at a speed of 6–9 kminm/h (30 min) followed by cool-down stretching (5 min). CCRT training (EG2): by experienced therapists at a ratio of one therapist to five participants, 30 exercise sequences that dynamically adjust the difficulty as the accuracy reaches 80%.	TAU	14 individuals withdrew for personal reasons (Control 1, CAE 6, AE 7). Reasons: discharge before the trial started (CAE 1, AE 2); worsened symptoms (Control 1, AE 1); unwillingness to rush to the training room within the agreed-upon time (CAE 1; AE 2); not being able to complete computer operations (CAE 3); without a specified reason (CAE 1; AE 2).	8 weeks	EG1: 5/week, EG2: 2/week	EG1: 45 min, EG2: 45 min	EG1: 50–70% HRR	Antipsychotics (FGA/SGA): EG1: 3/22, EG2: 2/24, CG: 3/28. Equivalent dose of chlorpromazine: EG1: 328.00 ± 96.91, EG2: 357.69 ± 102.66, CG: 374.19 ± 97.36	I: SCWT
[54] RCT	Yoga: Most of the yoga sessions were individual sessions (a maximum of three subjects were taught together in a few sessions). Most of the subjects finished 20 sessions in 6 weeks. Few subjects finished in 4 weeks.	Waitlist	Yoga group: One move to another place; one discontinued intervention. Waitlist group: One could not be contacted.	6 weeks (20 sessions)	4–5/week	60 min	Not reported	Antipsychotic: EG: 518.0 ± 329.05, CG: 434.0 ± 191.33	SC: SOCRATIS TofM: story-based tasks SP: social cue recognition test ER: TRENDS
[55] RCT	Treatment-as-usual plus aerobic walking (TAW): The target dosage of the structured walking program, based on the American College of Sports Medicine guideline. The participants walked for 30 min after a 5 min warm-up. The schedule was flexible, and the participants could decide the number of days they would attend to achieve a feasible goal of 150 min per week, for example, 50 min sessions three/week.	TAU	During the 12-week trial period, one participant in the TAU group dropped out due to personal issues; two in the TAW group lacked motivation and withdrew consent. Finally, 33 (97.1%) participants in the TAU group and 31 (93.9%) in the TAW group completed the 12-week trial and posttrial assessment.	12 weeks	5/weeks	30 min	30–40% HRR	Aripiprazole or Ziprasidone: EG: 18.20%, CG: 11.80%; Olanzapine or Clozapine: EG: 51.5%, CG: 50.00%; Other antipsychotics: EG: 30.30%, CG: 38.2%	WM: Digit Sequencing Task (BACS)
[56] RCT	Yoga: a specific yoga therapy module for schizophrenia, by a professional yoga therapist at the Advanced Center for Yoga in NIMHANS for a period of 1 month. It consisted of loosening exercises, breathing practices, Suryanamaskāra, sitting and supine and prone posture Āsanas, along with Prāṇāyāma and relaxation techniques.	Waitlist	In the yoga group, all 15 patients completed baseline and follow-up assessments. The 16 patients who dropped out in the waitlist group did so primarily due to logistic reasons.	1 month	Not reported	Not reported	Not reported	Not reported	ER: TRENDS
[48] RCT	Each session includes a 5 min warm-up and cool-down period, as well as stretching exercises. The walking duration gradually increases over the 12-week period. During weeks 1–2, veterans walked for 20 min per session; during weeks 3–4, they walked for 30 min per session; and from weeks 5–12, they walked for 40 min per session (the desired maximum outcome). The walking exercises were led by two research personnel trained on the study procedures by the PI and monitored for fidelity on a weekly basis.	Stretching exercise: 5 min warm-up and cool-down period of slow walking	One participant dropped out of the study after randomization and before completion of baseline testing, leaving 53 subjects available for data analyses.	12 weeks	3/week	40 min	60–70% HRR	Antipsychotic medication: atypical (EG: 83%; CG: 72%), typical (EG: 9%; CG: 16%)	WM: WAIS-IV letter-number sequencing test I: AX-CPT ER: Facial Emotion Identification Test TofM: The Awareness of Social Inference Test (TASIT–Part 2)
[57] RCT	Exercise intervention: One session, three phases: warm-up (15 min), aerobic exercise (30–40 min), and cool down (5 min). In the warm-up phase, the participants stretched and could talk to one another. In the aerobic exercise phase, the participants could choose which one activity they preferred: an ergometer and/or aerobics and/or Tai-chi.	TAU	The dropout rates in the TAU and exercise groups were 17.6% and 20%, respectively.	8 weeks	2/week	60 min	Mild intensity	FGA (mg): EG: 246.4 ± 358.4, CG: 87.5 ± 143.6 SGA (mg): EG: 802.3 ± 589.4, CG: 1000.00 ± 516.4 Antipsychotic (mg): EG: 1048.7 ± 846.4, CG: 1087.5 ± 632.9	CF: Cognitrax Basic Version
[59] RCT	Bike ergometer AET: Participants cycled consistently at lactate threshold intensity (2 mmol/L, slightly strenuous, talking possible). 5 min warm-up/cool down at 80% intensity. Lactate tests every 4 weeks, adjusted training intensity.	Exercises focused on flexibility, stability, balance, and relaxation. 5 min warm-up and cool-down at 80% intensity, without using lactate threshold for intensity definition.	59.55% of the AET group and 57.14% of the FSBT group dropped out of the study during the six-month active study phase	6 months	3/week	40 min 1–6° weeks, 45 min 7–12° weeks, 50 min 13–26° weeks	Slightly strenuous but should have still been able to talk without being short of breath.	Not reported	WM: Digit Span Test ER: Pictures of Facial Affect Recognition Test CF: TMT global
[58] RCT	Aerobic exercise: stationary bicycle ergometer. 20 min per session to a maximum of 45 min by increasing 5 min each week	Stretching and balance training	The final cohort of subjects who completed 12 weeks of treatment included nine in the AE group and six in the CON group.	12 weeks	3/weeks	20–45 min	50% HRR	Atypical Antipsychotics: EG: 14.00 ± 66.67, CG: 13.00 ± 76.47; Typical Antipsychotics: EG: 1.00 ± 4.76, CG: 2.00 ± 11.76; Both (Atypical and Typical): EG: 2.00 ± 9.52, CG: 1.00 ± 5.88; Antidepressants: EG: 6.00 ± 28.57, CG: 4.00 ± 23.53	WM: MCCB SC: MCCB
[60] Non-RCT	Cognitive training and aerobic exercise: exercise videos approved by a certified personal trainer. Calisthenics (e.g., lunges, squats, pushups) and simple movement sequences at several levels of intensity, without resistance training.	Neurocognitive training and social cognitive training. Bridging group: discussions about how computerized training exercise could help.	Attendance at the CT sessions, 90% of these in-clinic sessions. For the exercise groups at the clinic, 95% attendance. Adherence with the at-home exercise, 92%.	10 weeks	CG: 2/week EG: 2/week + 2/week	CG: 60 min EG: 150 min/week + 2 session (45 min) + 30 min at clinic	60–80% HRR	Second-generation antipsychotic medication: 11 oral risperidone, three long-acting injectable risperidone, two aripiprazole, two with supplemental quetiapine	WM: MCCB SC: MCCB
[61] RCT	Outdoor cycling: (1) 15 min for setting goals for the day and safety education, (2) 10 min to warm-up exercise, 40 min of bike training, 10 min of cool-down exercise, (3) 15 min for discussion on achievement of day.	Occupational therapy	OC and OT groups 13.3% and 20%, respectively. Dropout rates did not vary significantly across intervention groups [χ2(1) = 0.480, *p* = 0.488].	16 weeks	N. R.	40 min	Not reported	Second-generation antipsychotic and regular visits with the treating psychiatrist: all participants.	CF: WCST
[62] RCT	Aerobic exercise: individual and group programs by occupational therapists. In the group AE program, exercise videos. Squats, repeated lateral movements, arm and leg movements, and deep breathing.	TAU	The TAU + AE patients completed an average of 85.75% (mean = 20.58 sessions, standard deviation = 2.63) of the AE sessions over 12 weeks.	12 weeks	2/week	60 min	60–80% HRR	Antipsychotic	WM: BACS
[64] RCT	Endurance training: outdoor walking, Nordic walking, or running.	Occupational therapy	13 dropout = 10 patients because of early discharge from hospital and 3 patients because of various other reasons.	>4 weeks (22 sessions)	3/week	20–30 min	85% MHR	N.R.	WM: digit span of the German adaption of the revised Wechsler Memory Scale. CF: a computer-assisted card-sorting procedure (CKV).
[65] RCT	Guided intervention in physical exercise: walking (aerobic resistance).	Typical daily physical activity (e.g., shopping at the supermarket), without guidance or incentives.	Retention rate: 100%	12 weeks	3/week	60 min	64–76% MHR	N. R.	Inhibition: Stroop Color and Word test (SCWT). CF: Wisconsin Card Sorting Test (WCST); TMT-B WM: Verbal Fluency; digit span backwards subtest (WAIS-III).

AE = Aerobic exercise; AET = Aerobic endurance training; AX-CPT = AX-Continuous Performance Task; BACS = Brief Assessment of Cognition in Schizophrenia; CAE = CCRT combined with aerobic exercise; CCRT = Computerized cognitive remediation therapy; CNB = Hindi version of the Penn Computerized Neuropsychological Battery; CF = Cognitive Flexibility; CKV = Computergestütztes Kartensortierverfahren; EF = Executive Function; ER = Emotion Recognition; FGA = First-generation antipsychotics; HRR = Heart Rate Reserve; MCCB = MATRICS Consensus Cognitive Battery; MHR = Maximal heart rate; min = Minutes; NIMHANS = The National Institute of Mental Health and Neurosciences; PE = Physical exercise; RCT = Randomized Controlled Trial; SC = Social Cognition; SCWT = Stroop Color-Word Test; SGA = Second-generation antipsychotics; SVYASA = Swami Vivekananda Yoga Anusandhana Samsthana; SP = Social Perception; TofM = Theory of Mind; TAU = Treatment as usual; TASIT = The Awareness of Social Inference Test; TMT-B = Trail-Making Test Part B; TRENDS = Tool for Recognition of Emotions in Neuropsychiatric Disorders; WAIS-III = Wechsler Adult Intelligence Scale short form; Wisconsin Card Sorting Test (WCST); WM = Working Memory; YT = Yoga therapy.

**Table 4:** GRADE analysis of “should physical exercise be used versus control in schizophrenia?”.

Certainty Assessment							№ of Patients		Effect		Certainty	Importance
№ of Studies	Study Design	Risk of Bias	Inconsistency	Indirect Evidence	Imprecision	Other Considerations	Exercise	Control	Relative (95% CI)	Absolute (95% CI)		
Emotion Recognition												
4	RCT	Not serious	Serious ^a^	Not serious	Not serious	none	106	66	-	ES 0.299 (0.062–0.535)	⨁⨁⨁◯ Moderate ^a^	IMPORTANT
Working Memory												
8	RCT	Not serious	Not serious	Not serious	Not serious	none	122	106	-	ES 0.132 (0.005–0.523)	⨁⨁⨁⨁ High	IMPORTANT
Inhibition												
3	RCT	Not serious	Not serious	Not serious	Not serious	none	98	54	-	ES 0.156 (0.173–0.484)	⨁⨁⨁⨁ High	IMPORTANT
Cognitive flexibility												
4	RCT	Not serious	Not serious	Not serious	Not serious	none	130	123	-	ES 0.171 (0.245–0.586)	⨁⨁⨁⨁ High	IMPORTANT

^a^ of 4 studies, two have similar results in favor of the intervention but the other two have mixed results, one of them being in favor of the intervention and one of them against; CI = Confidence Interval. GRADE certainty of evidence rating: Each filled circle with a plus (⊕) typically represents a level of certainty (e.g., high, moderate, low, very low). An empty circle (◯) usually indicates a lower level or a deduction in certainty.

## Data Availability

Data will be made available on request.

## Supplementary Materials

The following supporting information can be downloaded at https://www.mdpi.com/article/10.3390/sports13040123/s1, Supplementary S1: Specific search strategy for each database; Supplementary S2: Full text articles decision; Supplementary S3: PRISMA checklist.
