# Supplementary material for: Effects of Physical Exercise on Executive Function in Schizophrenia: Systematic Review and Meta-Analysis
Source: Sports (Basel). 2025 Apr 16;13(4):123. doi: 10.3390/sports13040123 (PMC12030906; doi:10.3390/sports13040123)
Supplement: Supplementary file 1 [file sports-13-00123-s001.zip › Supplementary S1.pdf]

Supplementary S1: Specific search strategy for each database

Database	Search strategy
EBSCO	TX (“executive function*” OR “cognitive function*” OR cognition OR “inhibitory control” OR “inhibition” OR “interference control” OR “executive control” OR “working memory” OR “updating” OR “cognitive flexibility” OR “shifting” OR “switching” OR “social cognition” OR “emotion regulation” OR “emotion recognition” OR “decision making”) AND TX (sport OR “modified sport” OR fitness OR exercise OR “physical activity” OR athletics OR "resistance training" OR “sport practice” OR “mindful movements” OR “mindfulness practice*” OR “yoga” OR “team game” OR “soccer”) AND TX (schizoph* OR catatonia OR "schizoaffective disorder" OR "schizophreniform disorder" OR "schizoid personality disorder" OR "psychotic disorder" OR "schizophrenia spectrum and other psychotic disorders")
PubMed	(((((((((("executive function*"[Title/Abstract]) OR ("cognitive function*"[Title/Abstract])) OR (cognition[Title/Abstract])) OR ("inhibitory control"[Title/Abstract])) OR inhibition[Title/Abstract])) OR “interference control”[Title/Abstract])) OR “executive control”[Title/Abstract])) OR ("working memory"[Title/Abstract])) OR ("updating"[Title/Abstract])) OR ("cognitive flexibility"[Title/Abstract])) OR (shifting[Title/Abstract])) OR (“switching”[Title/Abstract])) OR (“social cognition”[Title/Abstract])) OR (“emotion regulation”[Title/Abstract])) OR (“emotion recognition”[Title/Abstract])) OR (“decision making”[Title/Abstract])) AND ((((((((sport[Title/Abstract]) OR ("modified sport"[Title/Abstract])) OR (fitness[Title/Abstract])) OR (exercise[Title/Abstract])) OR ("physical activity"[Title/Abstract])) OR (athletics[Title/Abstract])) OR ("resistance training"[Title/Abstract])) OR ("sport practice"[Title/Abstract]) OR ("mindful movements"[Title/Abstract])) OR ("mindfulness practice"[Title/Abstract])) OR ("yoga"[Title/Abstract])) OR ("team game"[Title/Abstract])) OR ("soccer"[Title/Abstract]))) AND (((((((schizoph*[Title/Abstract]) OR (catatonia[Title/Abstract])) OR ("schizoaffective disorder"[Title/Abstract])) OR ("schizophreniform disorder"[Title/Abstract])) OR ("schizoid personality disorder"[Title/Abstract])) OR ("psychotic disorder"[Title/Abstract])) OR ("schizophrenia spectrum and other psychotic disorders"[Title/Abstract]))
Scopus	(("executive function*") OR ("cognitive function*") OR (cognition) OR ("inhibitory control") OR (inhibition) OR (“interference control”) OR (“executive control”) ("working memory") OR ("updating") OR ("cognitive flexibility") OR (shifting) OR (switching) OR (“social cognition”) OR (“emotion regulation”) OR ("emotion recognition”) OR ("decision making")) AND ((sport) OR ("modified sport") OR (fitness) OR (exercise) OR (“physical activity") OR (athletics) OR (“resistance training”) OR (“sport practice”) OR (“mindful movements”) OR ("mindfulness practice”) OR (yoga) OR (“team game”) OR (soccer)) AND ((schizoph*) OR (catatonia) OR ("schizoaffective disorder") OR ("schizophreniform disorder") OR ("schizoid personality disorder") OR ("psychotic disorder") OR ("schizophrenia spectrum and other psychotic disorders"))
Web of Science	((TS = (“executive function*” OR “cognitive function*” OR cognition OR “inhibitory control” OR inhibition OR “interference control” OR “executive control” OR “working memory” OR “updating” OR “cognitive flexibility” OR shifting OR switching OR “social cognition” OR “emotion regulation” OR “emotion recognition” OR “decision making”)) AND TS = (sport OR “modified sport” OR fitness OR exercise OR “physical activity” OR athletics OR ""resistance training" OR “sport practice” OR “mindful movements” OR “mindfulness practice” OR yoga OR “team game” OR soccer)) AND TS = (schizoph* OR catatonia OR "schizoaffective disorder" OR "schizophreniform disorder" OR "schizoid personality disorder" OR "psychotic disorder" OR "schizophrenia spectrum and other psychotic disorders")
